# Histology of sheep temporal bone

**DOI:** 10.1590/S1808-86942011000300003

**Published:** 2015-10-19

**Authors:** Hormy Biavatti Soares, Luiz Lavinsky

**Affiliations:** 1 MD, Otorhinolaryngologist - Centro Otorrinolaringológico de Florianópolis, SC. Pesquisador, National Council of Scientific and Technological Development, CNPq; 2 Department of Otorhinolaryngology - University Hospital of Porto Alegre, Porto Alegre, RS; Graduate Program in Medicine: Surgery, Medical School - Federal University of Rio Grande do Sul, Porto Alegre, RS

**Keywords:** histology, ear, temporal bone, sheep

## Abstract

Previous studies suggest that there is an excellent correlation between the morphology and dimensions of ear structures in sheep and human beings.

**Aim:**

To analyze and describe the histology of structures inside the temporal bone in sheep.

**Material and Methods:**

A total of 307 slides obtained from vertical and horizontal sections of the temporal bone of eight sheep were analyzed. Structures were classified as similar or not similar to human structures, based on cellularity and histological architecture parameters.

**Study design:**

Experimental.

**Results:**

The study revealed similarities between sheep and humans in terms of type of epithelium, bone component, spaces in the auditory meatus, in addition to a marked histological resemblance of cellularity and that of the structures surrounding the ear. The main differences observed were the presence of an anatomic bulla, the absence of aeration in the mastoid and the inferior opening of the hypotympanum into the bulla in sheep.

**Conclusion:**

Based on these observations, it is possible to conclude that sheep represent an adequate option for training and research in otologic surgery.

## INTRODUCTION

Experimental research in otology used the most different species of animals, such as chinchilla, guinea pigs, rats, cats, dogs, monkeys, and others^1^, ^2^, ^3^, ^4^, ^5^, ^6^, ^7^, ^8^. The decision about which animal to be used, often times depends on the study goals. Among the criteria used to select a given animal, we have anatomical similarity, caging conditions, the animal's commercial availability and also the availability of reagents used to document inflammatory reactions.

In 1999, Lavinsky et al.^9^ published a study, without precedence, about sheep ears and their surgical aspects, stressing the great usefulness of this animal in the testing of complex surgical procedures. The group has done a number of morphometric studies on the middle ear structures of sheeps^10^, ^11^. These studies show the excellent relationship between the sizes of the temporal bones of sheep and humans.

The goal of the present study was to complement those studies, providing a histological description of the structures which make up the sheep ear.

## MATERIAL AND METHODS

We carried out a descriptive study of the temporal bones from eight *Corriedale* sheep. The vertical and horizontal cross-sections of the temporal bones from eight sheep yielded 307 slides. The procedures associated to the creation of these slides have been previously described^12^.

The present study encompassed only the histological analysis of slides and, therefore, it was not necessary to approve the protocol by the ethics committee. The original study protocol involving the animals was approved by the Ethics Committee of the institution where the study was carried out (protocol # 01090).

### Histological analysis

The slides were observed using a BX-60 Olympus microscope with a double binocular head (Olympus, Philadelphia, USA). The histological findings were described and recorded.

In the routine of describing the findings, the anatomical elements and accidents which are relevant for human otology were prioritized, according to the literature^12^, ^13^: external ear canal; middle ear; Eustachian tube; mastoid; pyramidal eminence; stapes muscle; tympanic membrane (tensor muscle and tensor muscle tendon); oval window; annular ligament; malleus ligament; facial nerve; endolymphatic duct; lateral ampullary nerve; vestibular nerve; utriculus; ossicles, stria vascularis; saccule; tympanic bulla; and cochlea.

In the description of histological elements, we recorded the intracavitary spaces and the types of tissue present. In a second procedure, we compared these elements and their corresponding counterparts in human beings, by means of a binomial decision which characterized the structures as being similar or not to those from human beings. The comparison standards utilized were cellularity and the architecture of the structures in a histological basis. We considered the elements which met both criteria, that is, those which presented the same type of cells in the element being analyzed and anatomical similarity (visual analysis).

### Image documentation

The images describing the histological architecture were obtained using the 900 series DF Vasconcelos surgical microscope, with a Nikon Coolpix 5.0 digital camera (Tokyo, Japan) fit to the microscope. The images which searched the tissues inwards and cellularity were obtained by an image capturing system coupled to a BX-60 Olympus microscope and specific software (ACDSeeView 4.0, ACDSee, Victoria, Canada). The magnifications utilized were 3, 5, 8, 13, 20, 25 and 40 X.

## RESULTS

**Figure 1:**
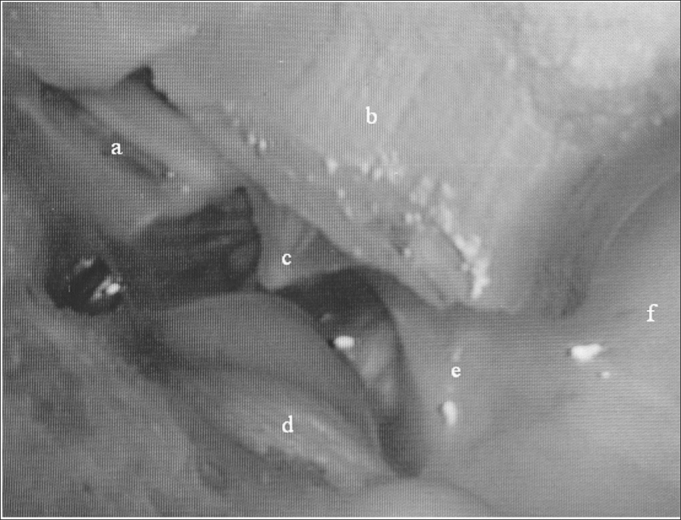
Medial wall view: a) epitympanic; b) external ear canal; c) tympanic membrane; d) Jacobson's nerve; e) hypotympanum; f) tympanic bulla.

In order to help understand the histopathology findings, Figure 1 depicts sheep temporal bone dissections. The main structures analyzed are described below.

## Bulla

Although absent in human beings, the tympanic bulla is useful for research purposes^2^, ^6^, ^14^, ^15^. In sheep, this structure is a broad and almost smooth cavity, of very slender walls. It is internally coated by a low columnar epithelium, forming only one row of cells. In certain cross-sections, one can see a sequence of pseudo cavities, restricted to the wall, which provide support to the bulla, without segmenting it at any point.

**Figure 2:**
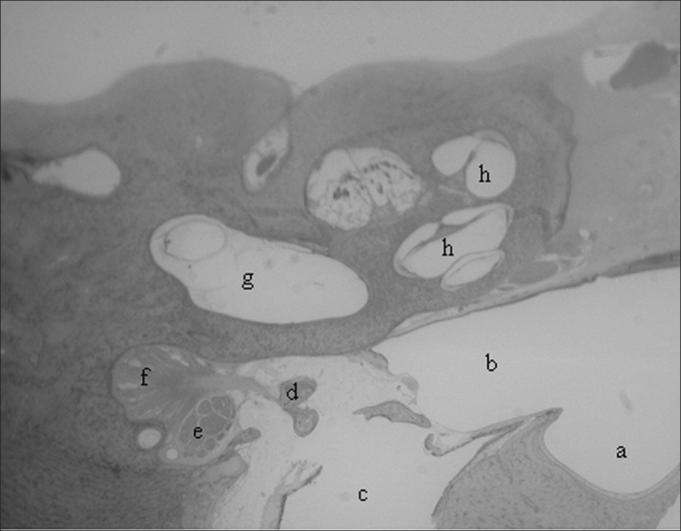
Low magnification cross-sections (3X): a) tympanic bulla; b) hypotympanum; c) external ear canal; d) stapes head; e) facial nerve; f) stapes tensor muscle; g) vestibule; h) cochlea.

The bulla is in contact with the cochlea, the hypotympanum, the Eustachian tube and the petrous portion of the temporal bone, and it borders the external auditory meatus, being separated from it by a fibro-cartilaginous tissue (Fig. 2).

## Cochlea

The cochlea is inserted in the temporal bone (*pars petrosa*), a large part of it is in contact with the bulla tympanic and the hypotympanum. It is wrapped in a cancellous bone tissue called modiolus, which contains a nerve ganglion, the spiral ganglion, forming a snail-like structure of bony walls. Internally, the cavity is coated by clear polygonal cells. Inside, there is a membranous portion with a cone inside. This cone divides the space, creating a triangle.

**Figure 3:**
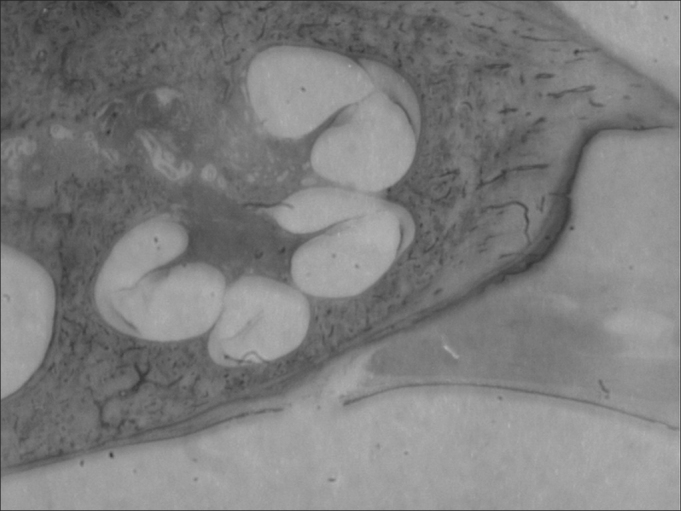
Cochlea (small magnification, 3 X).

In a cross-section, three portions of the triangle can be identified in relation to the bony space: a superior one, or scala vestibuli; a medium, or scala media; and an inferior, or a scala tympani. These names are due to the fact that the scala vestibuli opens up to a vestibule and the scala tympani communicates with the tympanic cavity, by means of the round window. The scala vestibuli and the scala tympani are filled up by perilymph and they are in contact with each other, in their ends, through the helicotrema, a small orifice in a portion strangled by the end of the scala media (Fig. 3).

## Organ of Corti

**Figure 4:**
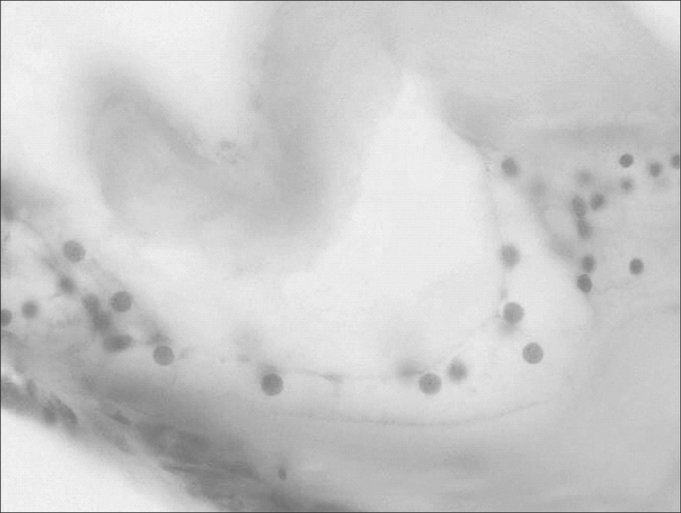
Organ of Corti (large magnification, 25 X).

The organ of Corti can be clearly outlined, with the tectorial membrane, the basilar membrane and the *stria vascularis* (Fig. 4).

## Mastoid

**Figure 5:**
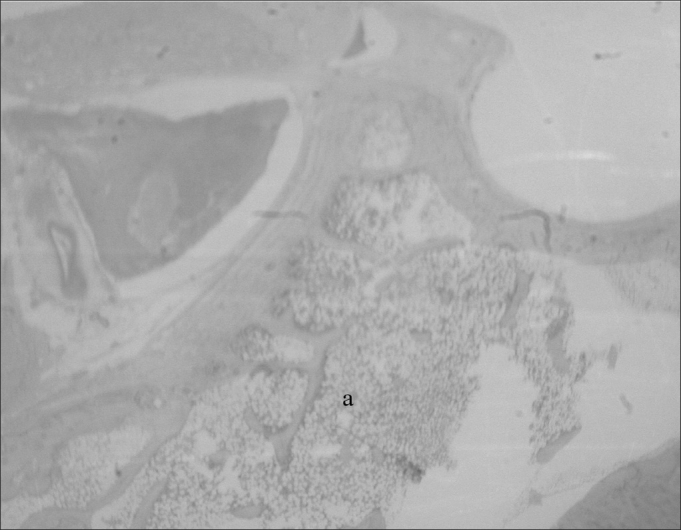
Mastoid: a) Cells filled up by hematopoietic tissue (small magnification, 3 X).

The sheep mastoid is a cancellous bone with trabeculae forming small cavities: the mastoid cells. These are filled up by fat and hematopoietic tissues - actually precursor cells of the granulocytic erythroid and megakaryocytic series, permeated by blood vessels (Fig. 5).

## Tympanic membrane

The tympanic membrane is extremely thin and seems to be internally coated by respiratory epithelium, and externally it is coated by stratified squamous epithelium. It does not have a fibrous middle layer and there is a central portion involving the manubrium. One can see that the malleus handle is inserted in the tympanic membrane, which inside has the same epithelium as the tube's proximal portion.

## Middle ear

**Figure 6:**
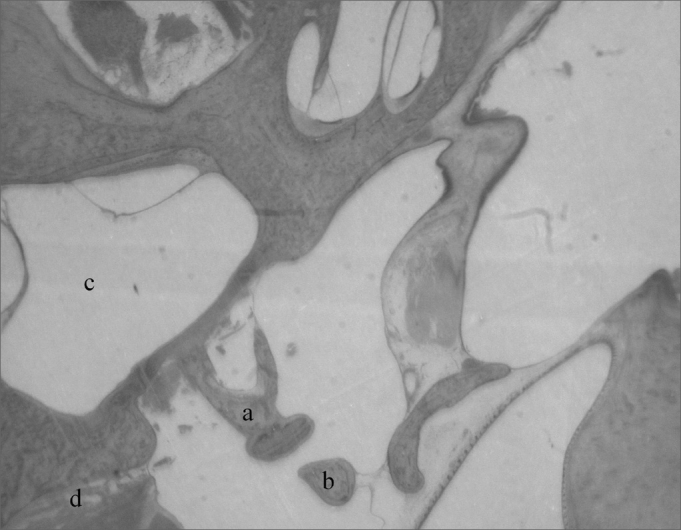
a) Stapes; b) long arm of incus; c) vestibule; d) tenor stapes muscle (small magnification, 3 X).

The middle ear is a very irregular cavity, with folds coated by respiratory epithelium. It has the ossicles, the tympanic membrane, and the space laterally limited by the membrane, inferiorly by the bulla and anteriorly by the tube's opening. It has two striated muscles: the tensor tympani muscle and the stapes tensor muscle, which are inserted in the malleus handle and in the stapes, respectively. It is also possible to clearly notice the stapes tendon inserted on the stapes head. The stapes muscle canal is commonly in communication with the fallopian canal, near the pyramidal eminence (Fig. 6).

## Auditory Tube

The auditory tube is coated by stratified columnar respiratory mucous-producing epithelium with cilia and some mucosal glands. Right below this epithelium, near the pharynx, there is fibro-cartilaginous tissue and a thin layer of bone tissue.

**Figure 7:**
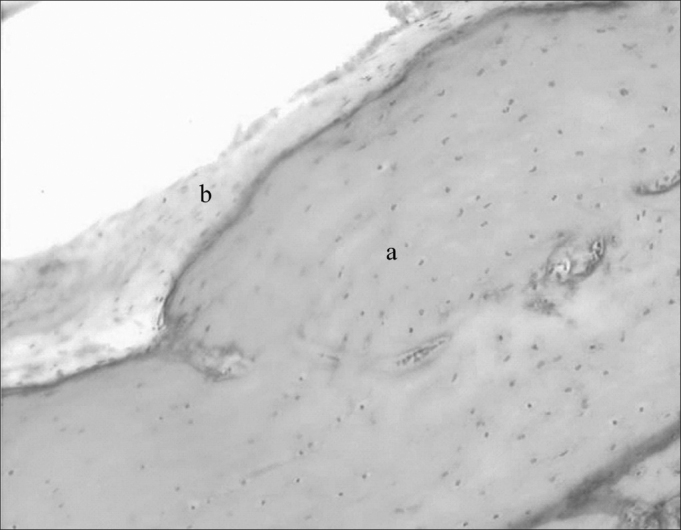
Auditory tube in large magnification (20 X): a) cartilaginous portion; b) respiratory epithelium.

In a cross-section below the middle ear the tube shows up with a large quantity of mucous glands, draining to its lumen and going to a more distal portion, which is the bulla with a small recess. At the final portion of the auditory tube, there is cartilage and a thin bone membrane (Fig. 7).

## External ear canal

With a 70 degree tilt in relation to the tympanic membrane, the external ear canal is coated by a hair-rich skin and by some few sebaceous and cerumen glands. In the more distal portion of the canal, we see surface granules, made up of keratohyalin.

The lumen of this canal is broad. The skin is very thin, with one single keratinized stratified cell layer. Below this layer, there is a thin layer of fibrous tissue and one of compact bone tissue. The fat tissue becomes more abundant the more distal one moves from the tympanic membrane.

## Facial nerve

In the facial nerve we see fibers and axon bodies. Most of its extension is associated with the horizontal semicircular canal. Its characteristic eosinophilic aspect shows up in the different cross-sections.

**Figure 8:**
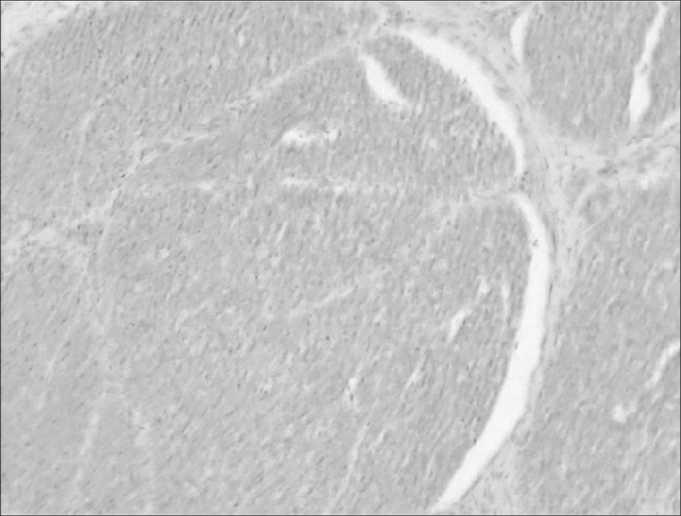
Axonal bundles of the facial nerve (characteristic eosinophilic aspect) (medium magnification, 13 X).

In the coronal sessions, we see a facial nerve constantly located next to a semicircular canal: the horizontal semicircular canal. On a cross section of a canal, it is formed by a thin layer of compact bone with a structure inside: the membranous labyrinth. The membranous portion has a simple squamous epithelium and connective tissue (Fig. 8).

## Ossicles

Looking at a cross-section made perpendicular to the malleus, this area is formed by bone and cartilaginous tissues, that is; bone calcification in an endochondral matrix. The malleus has more mass towards the proximal portion of the auditory tube when one moves upwards. On the incus there is a ligamentary fibrous tissue, forming a ligament with an upper portion and another one more posterior. The incus does not have the lenticular process.

The joint between the malleus and the incus has a cartilaginous portion with collagen fibers, forming an enarthrosis-type of joint.

The stapes is made up of a portion called footplate and two legs, or crura, the anterior and the posterior. Both are joined in the upper portion, forming the stapes' head. The head joins the incus through a joint ligament tissue, similar to what happens in humans.

## Vestibuli

**Figure 9:**
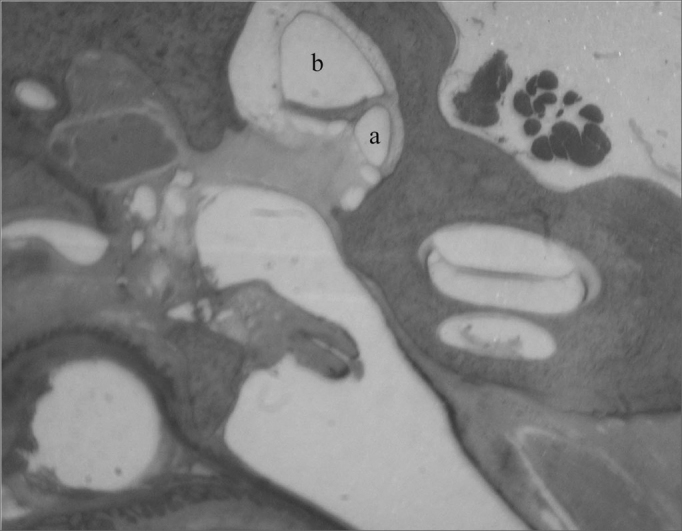
Vestibuli: a) saccule; b) utriculus (small magnification, 3 X).

The saccule and utriculus, perfectly formed, are both made up of a very thin membranous tissue, but a well-defined one. The oval window site has a clear window, just like that found in men, making up a niche for the oval window, which is made up by epithelial connective tissue. On it one can find the stapes footplate (Fig. 9).

The main histological findings are presented on Table 1.

**Table 1:** Description of the macroscopic and microscopic aspects of the temporal bone of *Corriedale* sheep (n=8)

Element	Macroscopic aspects	Microscopic aspects
External ear canal epithelium	Skin	Keratinized stratified squamous cells
Middle ear epithelium	Mucosa	Simple squamous epithelium with mucous producing cells
Tympanic bulla epithelium	Mucosa in a broad and smooth cavity	Simple ciliated epithelium with mucous producing cells
Mastoid cells	Trabeculation	Filled up by fat and hematopoietic tissue
Type of ossicle classification and otic capsule	Compact bone	Endochondral bone tissue
Cochlea	Has a snail shape with 2.5 turns	Bone tissue in three slides
Scala vestibuli	Present in a triangular shape	Membranous bone tissue
Scala media (cochlear duct)	Triangular shape	Coated by the stria vascularis, organ of Corti and mesothelial cells
Reissner membrane	Tilted portion of the scala media	Membranous bone tissue coated with simple squamous cell epithelium
Basement membrane	Horizontal portion of the scala media	Layers of connective tissue and extracellular matrix
Tectorial membrane	Horizontal direction on the organ of Corti	Amorphous/jelly structure, similar to the macula
Scala tympani		Membranous bone tissue
Round window/Round window membrane	Depression on the scala tympani at the level of the middle ear	Connective-epithelial membrane
Oval window/oval window niche/oval window membrane	Vestibuli depression	Connective-epithelial membrane
Pyramidal eminence	Hole in the posterior wall of the tympanic cavity	Endochondral bone tissue
Stapes muscle/stapes muscle tendon	Bony groove on the tympanic cavity posterior wall/muscle tendon	Skeletal muscle fibers/fibro-elastic connective tissue
Tensor tympani muscle/tensor tympani muscle tendon	From the cochleariform process to the malleus	Skeletal muscle fibers/fibro-elastic connective tissue
Annular ligament	Fibrous tissue separating the stapes footplate from the stapes on the oval window	Fibrous tissue of mesenchymal origin
Utriculus	In contact with the semicircular canals	Simple squamous cell epithelium with a thin layer of connective tissue
Saccule	In contact with the cochlear duct	Simple squamous cell epithelium with a thin layer of connective tissue
Organ of Corti	Standard shape	Deiter cells, Hensen cells, hair cells, internal and external sulcus cell, Claudius cells
Auditory tube (bony portion)	Long and narrow bony portion without relation with the internal carotid which opens up in the bulla	The mucosa coating its lumen is made up of low ciliated columnar epithelium
Auditory tube (cartilaginous portion)	Broad and extensive	Pseudo-stratified epithelium, columnar ciliated and more abundant globus cells

In comparison with human beings, the main differences seen were the following: the temporal bone does not have a well-outlined antrum, as it happens in human mastoids; and its mastoid cells are filled up by fat cells and hematopoietic precursor cells.

Table 2 shows the results achieved comparing the temporal bone histological characteristics between humans and sheep.

**Table 2:** Histologic similarities between the elements from the temporal bones of humans and sheepa.

Element	Similar to humans
External ear canal epithelium	yes
Middle ear epithelium	yes
Auditory tube epithelium	yes
Type of ossification	yes
Mastoid cells	no
Pyramidal eminence	yes
Stapes muscle	yes
Stapes muscle tendon	yes
Tympanum tensor muscle	yes
Tympanum tensor muscle tendon	yes
Oval window membrane	yes
Oval window niche	yes
Annular ligament	yes
Malleus ligament	yes
Facial nerve	yes
Endolymphatic duct	yes
Lateral ampullary nerve	yes
Vestibular nerve	yes
Utriculus	yes
Internal utricular crest	yes
Utricular macula	yes
Ossicles	yes
Stria vascularis	yes
Saccule	yes
Tympanic bulla	no
Cochlea	
2.5 turns	yes
Scala vestibuli	yes
Scala media	yes
Scala tympani	yes
Round window	yes
Organ of Corti	yes
Hair cells	yes
Basement membrane	yes
Tectorial membrane	yes
Reissner membrane	yes

Comparison patterns: cellularity and architecture in a histology basis. We considered similar those elements which met both criteria, that is, which had the same cell type in the element being analyzed and anatomical similarity (visual analysis).

The current analysis revealed that sheep has an important tissue similarity with human beings, which is adequate for experimentation and training in otologic surgery.

## DISCUSSION

When experimental studies are done with animal models, one important question is to what extent it is possible to extrapolate these observations to humans. According to Van der Ven et al., animal models must be biologically characterized and have their immune determinants well studied^7^. Thus, the detailed study of new animal models, as is the present case, provides an important contribution to research.

One difficulty in the comparison of ear structures from animals and human beings stems from the major variations in terms of structure sizes in human beings. Su et al. carried out cochlear aqueduct measures, round window membrane, and that of the facial recess in a large series of human temporal bones. Using an eye micrometer, we carried out very precise measures in histological slides. In all the measures we found huge individual variations, with large standard deviations^16^. In the present study, this difficulty was solved by utilizing the visual criterion to compare the histological elements between humans and sheep, without prioritizing morphometry. In fact, it was possible to notice an expressive homogeneity as to the architecture and cell content studied, with an almost absolute similarity between human and sheep histology at a microscopic level, and a high index of visual similarities for the more important structures. The main differences were the number of cell layers or rows.

As per described by Lim^17^, the middle year mucosa histology in humans is not different from the middle ear histology described for sheep. Both have a typical respiratory epithelium, made up especially by columnar epithelium with cilia and by secretory cells localized on a basal membrane above the lamina propria^17^. Thus, it seems that the main advantage of the sheep is its anatomo-histological similarities with the auditory organ of humans, which is undeniable. Moreover, since it is a middle-size animal, the size of the structures allows for the development and training of the classic and new surgical procedures^17^.

## CONCLUSIONS

Thus, it was possible to notice that sheep is especially adequate for otologic experiments. For instance, numerous authors mention the bulla as an element favoring the study of middle ear infectious processes, considering the ease of obtaining secretions from the middle ear and the direct access to this and other structures, such as the cochlea^2^, ^6^, ^14^, ^15^. In sheep, the bulla is broad; and in some sections in which we carried out measures with an ocular micrometer, we noticed an area measure even larger than the middle ear itself, a factor which increases the possibility of collecting a good sample of the effusion produced. The sheep bulla is easy to access in an antero-inferior plane and it bears a broad opening to the hypotympanum. Simultaneously, it has a communication with the external auditory canal, from which, however, it is separated by a fibrous connective tissue. Thus, sheep can be used for studying infectious processes.

Moreover, the fact that sheep has a communication between the auditory tube, the attic and the middle ear cavity, with portions very similar to those in humans, we consider that sheep will also be useful to study otitis media. Considering that many studies induce infections by blocking the auditory tube, we can stress that both in humans and in sheep, the tube has a coating epithelium with globus and ciliated cells.

In ear surgery, proper training of manual dexterity cannot be done directly in patients. One alternative is to do such training in temporal bones from human cadavers. Nonetheless, although the dissection of temporal bones from cadavers is mandatory for training, today there is a growing difficulty in obtaining such material. Moreover, as one tries to reproduce surgical techniques, besides the anatomy, one must consider other aspects such as anesthesia and surgical access. Thus, animal models have great importance in the training and research of otologic surgery. The histological similarities between sheep and human temporal bones prove the usefulness of this animal for such end.

Currently available light microscopy techniques, together with the proper processing of tissue, are able to unveil the elements which differentiate the temporal bones. With the magnification provided by the microscope, it is possible to observe its rich histology: the ossicles, the vessels, the nerves and sensorial structures, and other idiosyncrasies. Historically, it was Schucknecht who standardized the temporal bone study method, after introducing it in 1968, which opened up new horizons for the detailed analysis of tissues. The methodology advocated by Schucknecht was based on associating histology with the clinical manifestations presented by the patients^1^. Later on, other authors gave credibility to the method, which today is the gold standard in the study of tissue alterations.

Finally, considering that human temporal bones are difficult to come by to, sheep represent an excellent alternative for research projects and training in ear surgery.

## References

[1] Piltcher OB, Swarts JD, Magnuson K, Alper CM, Doyle WJ, Hebda PA. (2002). A rat model of otitis media with effusion caused by eustachian tube obstruction with and without Streptococcus pneumoniae infection: methods and disease course. Otolaryngol Head Neck Surg.

[2] Giebink S, Ripley ML, Shea DA, Wright PF, Paparella MM. (1985). Clinical-histopathological correlations in experimental otitis media: implications for silent otitis media in humans. Pediatr Res.

[3] Doyle WJ, Rood SR. (1980). Comparison of the anatomy of the eustachian tube in the rhesus monkey (Macaca mulatta) and man: implications for physiologic modeling. Ann Otol.

[4] Sade J, Carr CD, Senturia BH. (1959). Middle ear effusions produced experimentally in dogs: I. Microscopic and bacteriologic findings. Ann Otol Rhinol Laryngol.

[5] Claus GA. (1930). Experimentelle studien uber den verschuluss der tuba Eustachii beim Hunde. Hals Nasen Ohrenheildt.

[6] Browning GG, Granich MS. (1978). Surgical anatomy of the temporal bone in the chinchilla. Ann Otol Rhinol Laryngol.

[7] Van Der Ven LT, van den Dobbelsteen GP, Nagarajah B, van Dijken H, Dortant PM, Vos JG (1999). A new rat model of otitis media caused by Streptococcus pneumoniae conditions and application in immunization protocols. Infect Immun.

[8] Hellström S, Salén B, Stenfors LE. (1982). Anatomy of the rat middle ear. A study under the dissection microscope. Acta Anat (Basel).

[9] Lavinsky L, Goycoolea M, Ganança MM, Zwetch Y. (1999). Surgical treatment of vertigo by utriculostomy: an experimental study in sheep. Acta Otolaryngol.

[10] Seibel VAA. (2000). Estudo anatômico e morfométrico do osso temporal da ovelha com o objetivo da realizaçã o de cirurgia experimental e treinamento em cirurgia otológica [dissertation].

[11] Lavinsky L, Seibel V., Takasaka T, Yuasa R, Hozawa K (2001). Recent advances in otitis media.

[12] Paparella MM, Lamey SF, Goycoolea MV., Paparella MM, Shumrick A. (1991). Otolaryngology.

[13] Schuknecht HF. (1980). Methods of temporal bone removal and disposition. Advisory Committee of National Temporal Bone Banks Program of the Deafness Research Foundation.

[14] Goksu N, Haziroglu R, Kemaloglu Y, Rarademir N, Bayramoglu I, Akyilddiz N. (1992). Anatomy of the guinea pig temporal bone. Ann Otol Rhinol Laryngol.

[15] Meyerhoff WL, Giebink GS, Shea DA. (1984). Silent otitis media: an animal study. Ann Otol Rhinol Laryngol.

[16] Su WY, Marion MS, Hinojosa R, Matz GJ. (1982). Anatomical measurements of the cochlear aqueduct, round window membrane, round window niche, and facial recess. Laryngoscope.

[17] Lim DJ. (1979). Normal and pathological mucosa of the middle ear and eustachian tube. Clin Otolaryngol Allied Sci.

